# The Self-Prioritization Effect: Self-referential processing in movement highlights modulation at multiple stages

**DOI:** 10.3758/s13414-021-02295-0

**Published:** 2021-04-16

**Authors:** Clea Desebrock, Charles Spence

**Affiliations:** Crossmodal Research Laboratory, Department of Experimental Psychology, University of Oxford, Oxford, OX2 6GG UK

**Keywords:** Self-referential processing, Self-prioritization, Arm movements, Visual feedback, Approach motivation

## Abstract

A wealth of recent research supports the validity of the Self-Prioritization Effect (SPE)—the performance advantage for responses to self-associated as compared with other-person-associated stimuli in a shape–label matching task. However, inconsistent findings have been reported regarding the particular stage(s) of information processing that are influenced. In one account, self-prioritization modulates multiple stages of processing, whereas according to a competing account, self-prioritization is driven solely by a modulation in central-stage information-processing. To decide between these two possibilities, the present study tested whether the self-advantage in arm movements previously reported could reflect a response bias using visual feedback (Experiment 1), or approach motivation processes (Experiments 1 and 2). In Experiment 1, visual feedback was occluded in a ballistic movement-time variant of the matching task, whereas in Experiment 2, task responses were directed away from the stimuli and the participant’s body. The advantage for self in arm-movement responses emerged in both experiments. The findings indicate that the self-advantage in arm-movement responses does not depend on the use of visual feedback or on a self/stimuli-directed response. They further indicate that self-relevance can modulate movement responses (predominantly) using proprioceptive, kinaesthetic, and tactile information. These findings support the view that self-relevance modulates arm-movement responses, countering the suggestion that self-prioritization only influences central-stage processes, and consistent with a multiple-stage influence instead.

**Supplementary Information:**

The online version contains supplementary material available at 10.3758/s13414-021-02295-0.

Across a diverse range of experimental tasks, it has been demonstrated that processing stimuli associated with the self can modulate attention (Sui et al., 2009), perception (Golubickis et al., 2017; Sui et al., 2012), memory (Rogers et al., 1977; Turk et al., 2008), and decision-making (Liu et al., 2016). Studies using a perceptual-matching task to examine the Self-Prioritization Effect (SPE; Sui et al., 2012) have demonstrated that effects of self-relevance can be dissociated (at least in part) from those of other response-facilitating factors, such as stimulus familiarity (Sui et al., 2012; Woźniak & Knoblich, 2019), stimulus reward value (e.g., Qian et al., 2019; Yankouskaya et al., 2017), emotional valence (Schäfer et al., 2020; Stolte et al., 2015), and semantic elaboration (Sui & Humphreys, 2013). Distinct neural circuitry has also been demonstrated to underpin the self-advantage in the matching task (Sui et al., 2013; Yankouskaya et al., 2017). Certain researchers have proposed that self-relevance can operate across multiple stages of information processing (Humphreys & Sui, 2016; Sui & Humphreys, 2015).

## Self-prioritization: The matching task

Sui et al.’s (2012) shape–label matching task has become something of a standard in the literature to investigate effects of self-relevance. The use of neutral shapes rather than the traditional own name, face, or other self-related stimuli enables researchers to examine the influence of self-relevance without introducing those confounds associated with previous studies—namely, stimulus familiarity and overlearning. In a prototypical task, participants are instructed to associate social labels (e.g., self, stranger, friend) with neutral geometric shapes (e.g., a circle, a square, a hexagon). They then carry out a matching task in which they have to judge whether sequentially presented shape–label pairs match the designated associations or not. Greater accuracy and shorter RTs are robustly found in the self-associated shape–label matching condition (Golubickis et al., 2017; Hu et al., 2020; Humphreys & Sui, 2016; Woźniak & Knoblich, 2019).

The behavioural advantage for self in the task has been shown to correlate with functional connectivity between the ventral medial prefrontal cortex (vmPFC; linked to a self-representation) and the posterior superior temporal sulcus (pSTS; linked to social attention) and is thought to be modulated by top-down attentional control (exerted over earlier visual regions by dorsolateral prefrontal cortex and intraparietal cortex; Humphreys & Sui, 2016; Sui et al., 2013). Functional connectivity between the vmPFC and a classic WM region (frontoparietal cortex) has also been demonstrated to underpin self-prioritization in a spatial WM task (Yin et al., 2021). Based on a wide range of evidence across tasks, it has been proposed that self-relevant stimuli activate a self-representation in the vmPFC which modulates responses by functionally coupling with distinct domain-specific regions associated with different components of the self. Thus, self-relevance can modulate multiple stages of information processing (Humphreys & Sui, 2016; Sui & Humphreys, 2015).

## Self-prioritization: Influence at multiple-stages

In line with effects of self-relevance more widely, self-prioritization is thought to influence multiple stages of information processing within the matching task—the allocation of attention, memory (the retrieval of a self-representation), and decision-making processes (Humphreys & Sui, 2016; Liu et al., 2016; Sui & Humphreys, 2015). Other research suggests that self-prioritization in the matching task may influence motor-related processes as well. In an action-related adaptation of Sui et al.’s (2012) matching task, Frings and Wentura (2014) instructed their participants to associate an arm movement (moving a cursor with a mouse up, down, left, or right on a screen) with the self and other labels. A directional cursor indicated to participants which of the arm movements to execute. On reaching the side of the screen, the participants had to judge whether the label that appeared matched the allocated arm-movement or not by pressing one of two mouse buttons. Judgements were faster and more accurate in self-associated trials. Participants’ arm-movements terminated before a judgment response was made, thus, the authors demonstrated a *motor-related SPE—*a prioritization effect in matching a self-associated label and *action representation*. Frings and Wentura’s findings suggested that self-associated movements encoded at a ‘conceptual level’ (p. 1740) may be accessed preferentially in the task (perhaps at the level of internal verbal description or as motor imagery). The authors did not, however, examine whether self-relevance could modulate the overt arm-movement response itself. In contrast, Desebrock et al. (2018) investigated whether self-relevance could modulate both the initiation and execution^1^ of arm-movement responses in Sui et al.’s (2012) matching task. In line with previous work, an SPE was demonstrated in the initiation of responses. In a novel result, the authors also found that self-relevance modulated the overt movement response.

## Self-prioritization: A central-stage influence

Contrasting with the view that self-relevance can modulate multiple stages of information processing, a growing number of researchers have suggested that self-referential and other-referential processing may only be distinguished in later-stage, higher-order, cognitive processes (Miyakoshi et al., 2007; Siebold et al., 2015; Stein et al., 2016). These studies ruled out a perceptual modulation by self-relevance, but, in line with tradition in the literature, modulation of the motor stage was not considered. In contrast, in a recent study aimed at pinpointing the processing locus of the SPE, Janczyk et al. (2019) used Sui et al.’s (2012) matching task within the context of a dual-task Psychological Refractory Period (PRP) paradigm (Pashler, 1994; Welford, 1952) and found that self-prioritization only influenced central-stage processes.^2^

## Self-relevance and the motor stage

**Fig. 1:**
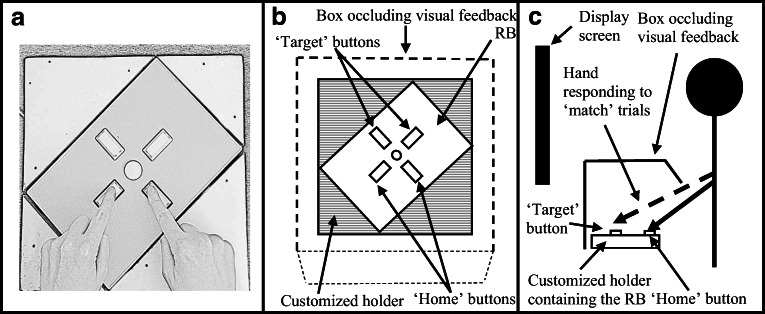
Schematic of Experiment 1 task apparatus and setup. RB = response box. **a** Aerial view of the RB and customized holder with participant holding down the ‘home’ buttons. The participant’s right hand moves to the top right (target) button, their left-hand to the top-left (target) button (box occluding visual feedback not shown). **b** Aerial view with box occluding visual feedback shown. **c** Cross-sectional view

Across three experiments, Janczyk et al. (2019) ruled out an SPE in perceptual processes. In their fourth experiment, the authors also found that the motor stage did not contribute to the SPE. The authors note, however, that this finding was “only preliminary given that [their participants made] discrete keypress responses, instead of, for example, continuous mouse movements” (Janczyk et al., 2019, p. 1080). These findings contrast with those reported by Desebrock et al. (2018), noted above. Instead of making key-press responses, Desebrock et al.’s participants released a ‘home’ button (measuring RT from stimulus onset up to movement onset), and executed a short arm movement to press a target key positioned in front of them a short distance away (measuring MT from the button release; see Figs. 1 and 5; Barton et al., 2020; Houlihan et al., 1994; Jensen & Munro, 1979; Praamstra et al., 2014). The authors were thus able to measure the overt movement stage of the response separately from the RT. They found that MTs were shorter and a higher-proportion of the movement responses were correctly completed. In Janczyk et al.’s study, movement completion accuracy performance was at ceiling for the button-press responses, but these authors ruled out in their preliminary finding that duration of the motor-stage was modulated.

## Self-relevance and the motor stage: An explicit response bias

If self-relevance can modulate the motor-stage of responses in the matching task, this would provide further support for the contention that self-relevance influences multiple stages of information processing. One limitation of Desebrock et al.’s (2018) study, however, is that the authors could not rule out that other factors may (part) account for the movement modulation. Notably, the *use* of visual information during the execution of the responses in the matching task, for example, was one of the salient differences between the studies reported by Janczyk et al. (2019) and Desebrock et al. Janczyk et al. comment that the motor stage of responses may be modulated in task responses using, for example, continuous mouse movements. Visual information pertaining to hand or target position was not relevant to, or requisite for, completing the discrete button-press responses in Janczyk et al.’s study (albeit that visual feedback was available in the sense that participants could see their fingers resting on the keys). By contrast, Desebrock and colleagues used rapid-aiming arm movements through a travel distance of 14 cm to a target button. It may be that effects of self-relevance in movement execution operate exclusively through visual-feedback-driven processes or their integration with other sensory information in the planning and/or execution of movements. If so, this may be through automatic processes (whether top down or bottom up; Gaspelin & Luck, 2018), constituting a genuine modulation by self-relevance of, for example, sensorimotor feedforward planning (see General Discussion). However, it also leaves open the possibility that a form of explicit, decisional response bias could have been operating during the execution of the overt movement. Decisional processes have been shown to leak into, and thereby influence, movement responses. For example, modulations of movement in mouse-tracking studies are thought to reflect changes of mind on the part of the participant (Grage et al., 2019). Notably, self-ownership prioritization—a related phenomenon—has been found to constitute a top-down decisional response bias (Golubickis et al., 2018). In Desebrock et al.’s (2018) task, the button being released (the choice) indicated the motor decision (effector and target) with no possibility of correction once released. Response selection processes thus occurred before the onset of the movement (Rubichi & Pellicano, 2004; Scorolli et al., 2015). However, using the opportunity provided by a *non*-ballistic task response (Glover, 2004; Khan et al., 2006), participants might still make explicit adjustments to the movement using visual feedback during its execution to favour the self-related response.

## Self-relevance and the motor stage: Affective evaluation processes

In addition to reflecting a form of top-down decisional bias, modulations of the movement response could be driven by automatic processes that may not pertain specifically to those of self-relevance. Effects of self-relevance in arm-movements could potentially be driven by differences in *approach-avoidance motivation* (Elliot, 2006; Kozlik et al., 2015; Solarz, 1960). The evaluation of appetitive or positive stimuli is thought to activate affective stimulus–response (S–R) compatibility mechanisms automatically which facilitate (e.g., arm-) movements that serve to *visibly* decrease the distance between the self and these stimuli (Kozlik et al., 2015; Krieglmeyer et al., 2013; Markman & Brendl, 2005; Piqueras-Fiszman et al., 2014; Seibt et al., 2008). Perceivable (i.e., visible) action effects (in terms of distance regulation between the participant and the stimuli) are thought to be a precondition for automatic affective S–R compatibility effects to arise (Kozlik et al., 2015; Krieglmeyer et al., 2010; Rougier et al., 2018; van Dantzig et al., 2008). Furthermore, self-associated stimuli may be automatically evaluated positively even if affective evaluation happens to be irrelevant to the task (Krieglmeyer et al., 2010; Stolte et al., 2015). In previous studies, the effects of positive emotional valence have been dissociated from those of self-relevance (e.g., Li et al., 2019; Stolte et al., 2015). On the other hand, whole arm-movement responses were visibly executed toward the stimuli in Desebrock et al.’s study, in contrast to key-press task responses in previous studies, and in Janczyk et al.’s (2019) research. The latter studies thus left the visible distance between the hand and the stimuli unchanged, and so were comparatively ‘approach neutral’ relative to Desebrock et al.’s task responses. Desebrock et al.’s task response and the availability of visual feedback may thus have activated affective S–R compatibility mechanisms in the context of Sui et al.’s task.

## Methodological rationale and hypotheses

Visual-specific (unimodal visual) processing and integrated visual and proprioceptive feedback are used to estimate hand position in arm-movement responses, while visual feedback is exclusively used to estimate target position (Gallivan et al., 2018; Krüger & Hermsdörfer, 2019; Scott, 2016). Therefore, in Experiment 1, visual feedback pertaining to both the hand and the target position was occluded in a task setup modelled on Desebrock et al.’s (2018) study (see Fig. 1) to test whether the advantage for self in arm movements was contingent on a modulation of these processes. In addition, removal of visual feedback would remove those ‘visible action effects’ thought to activate the automatic approach motivation that facilitates arm movements. The movement travel distance was also substantially shortened to 6 cm (as compared with 14 cm in Desebrock et al.) in order to elicit fast reactive (ballistic) movement responses (Glover, 2004; Khan et al., 2006). By such means, it was possible to determine whether an explicit decisional response bias (acting through online control using visual feedback) or affective evaluation processes could account for the modulation of movement. It was therefore hypothesized that if the self-advantage in Desebrock et al.’s (2018) task responses was not contingent on these processes, an advantage for self should arise in Experiment 1 (in terms of shorter MTs and a higher or equivalent proportion of correctly completed movement responses). An advantage for self would further suggest that self-relevance can modulate movement responses using only proprioceptive, kinaesthetic, and tactile information.

In Experiment 2*,* the participants executed their movement responses sideways, away from both the stimuli and their own body. In order to amplify potential avoidance motivation effects, visual feedback and the original movement travel distance (Desebrock et al., 2018) were reintroduced. Thus, executing sideways movement responses *visibly increased* the distance between the participant’s hand and the task-relevant stimulus, while being represented as ‘away’ from the stimuli and the body. Therefore, if the facilitation in responses to self-associated stimuli in Desebrock et al.’s task was solely underpinned by an automatic affective S–R mechanism, self-associated responses in Experiment 2 should be relatively disadvantaged, and responses to more negatively evaluated stranger-associated stimuli should be relatively facilitated. Therefore, if automatic affective S–R compatibility effects do not account for the self-advantage in Desebrock et al.’s study, a self-advantage should also arise in responses directed away from the stimuli and participant’s body.

To further test whether effects may be part-dependent on these processes, we also conduct a preliminary analysis comparing the self-advantage across the present study experiments and Desebrock et al.’s (2018) study to assess whether the self-advantage was reduced by the present study manipulations (see Supplementary Materials).

## Experiment 1

### Method

The effect size for the self-advantage in MT in Desebrock et al.’s (2018) study was large (paired-samples *t* test; *dz* = 2.46). However, occluding visual feedback might considerably reduce or extinguish the self-bias. Therefore, in order to allow for the detection of a smaller (medium-sized) effect (*dz* = 0.50), with a probability of 1 − β = .80, and an alpha value of .05, a minimum sample size of 34 participants was required (Faul et al., 2009).

#### Participants

Thirty-four right-handed participants (15 males, ages 18–40 years, mean age 24 ± 5.55 years) with normal or corrected-to-normal vision took part in Experiment 1. They were recruited via the Oxford University Research Participation Scheme and online university-group social media. They received course credit or monetary reimbursement for their time and effort. All of the participants completed a written consent form approved by the University of Oxford Central University Research Ethics Committee (MS-IDREC-R49190-RE002). One participant was excluded due to equipment failure, one for not completing the session, four for obtaining less than 55% correct in two or more conditions, and one constituted a multivariate outlier (Mahalanobis distance test, *p* < .01; Mahalanobis, 1930). The data from the remaining 27 participants (13 males, ages 18–37 years, mean age 23.56 ± 4.97 years) were included in the final analysis. The effect size detectable with 27 participants, an alpha value of .05, and a probability of 1 − β = .80, was *dz* = 0.57 (G*Power 3.1 program; Faul et al., 2009).

#### Apparatus and stimuli

The experiment was conducted on a PC with a 23-in. LCD monitor (1,920 × 1,080 pixels at 60 Hz refresh rate) using E-Prime software (Version 2.0). A Cedrus RB-530 response box recorded home-button-releases (measuring RT) and target-key-press (measuring MT) responses. The response box was positioned in front of a PC monitor. A cardboard box was placed over the response box, occluding the participant’s hands from direct sight. The response box was placed inside a custom-built wooden holder such that the ‘home’ and ‘target’ buttons were 6 cm apart (see Fig. 1a).

The stimuli consisted of two geometric shapes from the following set (circle, square, triangle, hexagon, pentagon, and octagon, each subtending 3.2 × 3.2 deg. of visual angle) and two self–other word labels (‘yours’, ‘theirs’, subtending a visual angle of 3.1 × 1.6 deg.). These stimuli/labels were counterbalanced across participants following a Latin Square design. Shape–label pairs (a geometric shape and personal label) were presented against a grey background in the centre of the PC screen. The shape was positioned above (and the label below) a red fixation cross (1.4 × 1.4 deg. of visual angle).

One consideration with regard to the use of the self- and stranger-associated labels, ‘yours’ and ‘theirs’, was that word concreteness has been shown to give rise to SPE-like prioritization effects, although effects of self-relevance go beyond those of word concreteness (Wade & Vickery, 2017). Previous studies have used a range of different labels to denote oneself and a stranger in Sui et al.’s (2012) matching task (e.g., you, self, I, stranger, other; Frings & Wentura, 2014; Golubickis et al., 2017; Hu et al., 2020; Sui et al., 2012). The well-established database of concreteness ratings for 40,000 English lemmas (Brysbaert et al., 2014) provides the following ratings for: ‘You’ (*M* = 4.11, *SD* = 1.22), ‘Self’ (*M* = 3.13, *SD* = 1.71), ‘I’ (*M* = 3.93, *SD* = 1.44), ‘Stranger’ (*M* = 3.76, *SD* = 1.39), ‘Other’ (*M* = 2.04, *SD* = 1.22). Notably, ‘Yours’ (*M* = 2.14, *SD* = 1.33) and ‘Theirs’ (*M* = 2.40, *SD* = 1.40) attracted among the lowest ratings for concreteness, and, importantly, very similar ratings to each other. These labels also had the advantage of being equivalent in length and having the same number of syllables.

#### Procedure and tasks

**Fig. 2:**
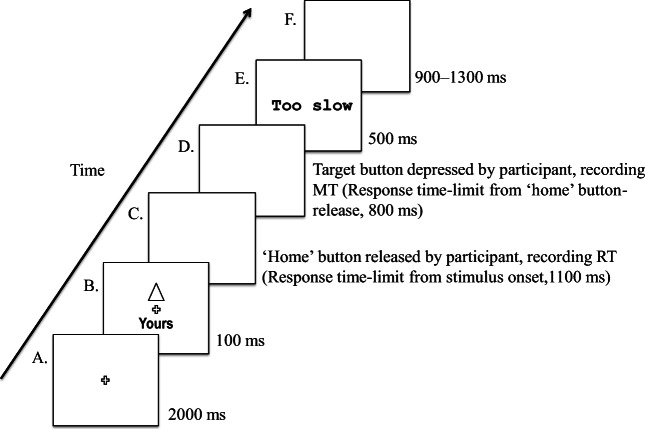
Schematic overview of a trial sequence (stimuli not to scale). **a** Fixation cross presented at the start of each block. **b** Stimulus onset. **c** Blank slide. **d** Blank slide **e** Onscreen feedback—“Correct”/“Incorrect”/“Too slow.” **f** Intertrial intervals generated at random. RT response time-limit = the time limit measured from stimulus onset within which a participant had to select their response and initiate the onset of the movement by releasing the ‘home’ button. MT response time-limit = the time limit measured from the release of the ‘home’ button within which a participant had to complete their movement response by depressing the ‘target’ button

The participants were instructed (via on-screen text) to associate one geometric shape with ‘self’ (specifically, as ‘yours’; e.g., ‘the square is yours’) and a second shape with ‘a stranger’ (as ‘theirs’; e.g., ‘the circle is theirs’) and to memorize these pairings. Following this, the participants completed the ‘matching’ task. The participants held two response-box buttons down with their index fingers before the first trial, and did so continuously throughout the task, except when making a response. To make a response, the participants released a response-box button by lifting an index finger and moving the hand forward to depress a target key with that index finger. The participants were instructed to make their response to the stimuli as rapidly and accurately as possible. Right-hand (i.e., dominant-hand) responses were made for those shape–label pairs participants judged as matching, and left-hand responses for those pairs judged to be mismatching. In our previous study (Desebrock et al., 2018), trial type (matching, mismatching) and assigned response options (using dominant or nondominant hand) were counterbalanced across two testing sessions per participant, and no interaction between hand (left, right) and association (self, stranger) in RT or MT was found. However, movements in the nondominant hand were slower, and there was an interaction across hands in sensitivity (*d'*; see pp. 263–264). These findings are consistent with established differences in preparatory and motor control mechanisms and associated brain activation across dominant/nondominant hand-motor networks (Babiloni et al., 2003; Dirnberger et al., 2011; Olex-Zarychta & Raczek, 2008; Poole et al., 2018; Sainburg, 2016). Furthermore, in the present study, only the match-trial data is analyzed because only match-trial responses index self-associated and stranger-associated processing (see Design and Data Analysis section). Mismatch trials are essentially fillers, and match and mismatch trials are typically analyzed separately (e.g., Janczyk et al., 2019; Sui & Humphreys, 2017; Woźniak et al., 2018). Therefore, participants in the present study made matching-trial responses with their dominant hand so that effects of self-related versus stranger-related responses could be compared without having to pool dominant and nondominant hand responses, consistent with previous studies using the dominant hand to make match-trial responses (e.g., Sui et al., 2012). Figure 2 provides a schematic representation of the matching task.

Preceding the main task, there was a practice block of 24 trials with the performance-accuracy threshold set at 60%. Participants repeated the practice block until this threshold was achieved. The main task consisted of four blocks of 80 trials separated by 8,000-ms breaks, with each condition randomly generated with an equal number of presentations (80 trials per condition). The participants were informed of their overall accuracy at the end of each block of trials.

#### Design and data analysis

There were two within-participants factors, each having two levels: association (self, stranger) and matching condition (matched, mismatched). There were four main output measures: reaction time (RT; measured from stimulus onset to the release of the response-box button), movement time (MT; measured from the response-box button release to the depression of the target key); proportion of correct response initiations, and proportion of correct arm-movement responses (successfully hitting the target key). An error in movement response completion consisted in not depressing the target key (missing or not landing squarely on the key) or hitting the incorrect target key, following a correct initiation response. Movement errors consisting of hitting the incorrect target key were negligible in the stranger-match (<0.5% / ~0.3%) and self-match (<0.1% / ~0.04%) conditions. All remaining errors consisted of a failure to correctly complete the movement response and hit the target key.

As noted in the Procedure and Tasks section, different responses are made to the two different types of stimuli (matching, mismatching; Janczyk et al., 2019; Sui & Humphreys, 2017; Woźniak et al., 2018). Responses in match trials index self-associated and stranger-associated processing (from which the self-bias measure is also calculated; e.g., Sui & Humphreys, 2017) and was the comparison of interest for the present study. In contrast, mismatch trials are typically treated as filler trials. They are a combination of self-associated and stranger-associated stimuli, and so self-related versus stranger-related processing in these trials cannot be disentangled in behavioural paradigms. Therefore, analysis of RT, MT, response initiation accuracy, and movement completion accuracy was carried out on the match-trial data.

Following previous work (Desebrock et al., 2018; Sui et al., 2012; Sui & Humphreys, 2017), a signal detection approach was also used to calculate an index of sensitivity (*d* prime; *d*′; Green & Swets, 1996). Hits were coded as *yes* responses to match trials, and false alarms were coded as *yes* responses to mismatch trials with the same shape; thus, sensitivity scores were derived from right (match)-hand responses only (namely, the same effector).

A normalized self-bias score was also calculated (following previous work; e.g., Sui & Humphreys, 2017) for RT, MT, and the proportion of correctly initiated and executed responses. This provides an index of the relative magnitudes of the difference in performance between self-associated and stranger-associated matched trial responses, and is given by the formula: “(stranger − self)/(stranger + self)” for RT and MT; and: “(self − stranger)/(stranger + self)” for self-bias in proportion of correct scores.

**Fig. 3:**
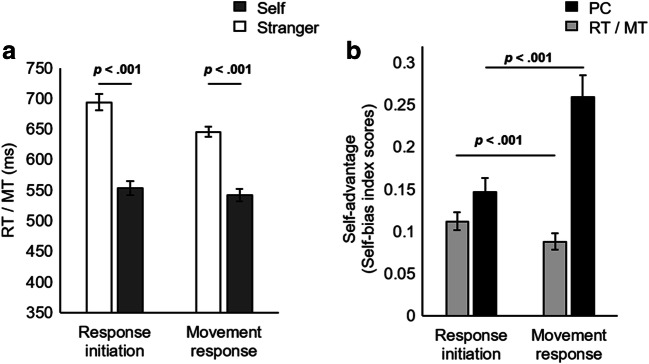
**a** Experiment 1. Mean RT (reaction time) and MT (movement time) as a function of association (self vs. stranger) in the shape-label matching trials. **b** Mean normalized self-bias index scores (magnitude of the self-advantage) in RT, MT, and proportion correct (PC) as a function of response stage (response initiation vs. movement response) in the shape-label matching trials. Error bars represent standard error

Only correct response initiations (RTs) were analyzed. RTs above or below 2.5 standard deviations from individual means were excluded, eliminating <2% (130) of the trials. Similarly, only correct movement responses (MTs) following a correct initiation response were analyzed. MTs greater than 2.5 standard deviations above individual means were excluded, eliminating <1% (41) of the trials. Overall, RT analyses were carried out using 81% of the trials, and MT analyses using 71%. Effect sizes were calculated using Cohen’s *dz* for *t* tests (Cohen, 1988; Lakens, 2013). Means and standard errors for self-associated and stranger-associated matching conditions are visualized in Fig. 3 (see Appendix, Table 1, for means and standard deviations of RT, MT, and proportion correct for response initiation and movement execution in both match and mismatch trials).

In the Supplementary Materials*,* we also report a preliminary analysis (ANOVA) comparing the self-bias in RT, MT, and movement completion accuracy across the present study experiments and Desebrock et al.’s (2018) study. An advantage for self in Experiments 1 and 2 would suggest that the self-advantage was not solely contingent on an explicit decisional response bias (acting through online control using visual feedback) or affective evaluation processes. However, if the self-advantage in the present study experiments was reduced as compared with Desebrock et al. (2018), this would suggest that the self-advantage might be part contingent on these processes (see Supplementary Materials, Table S1, for means and standard deviations of RT, MT, and proportion of correctly completed movement responses, with standard deviations, as a function of association [self vs. stranger] in the match condition for Experiments A1, 1, and 2).

### Results

#### Response initiation

##### Reaction time (RT)

A paired-samples *t* test revealed a significant difference between the RTs for self-associated match versus stranger-associated match trials, *t*(26) = 10.57, *p* < .001, *dz* = 2.03; self-associated match responses were initiated more quickly. Response initiation accuracy. One nonextreme outlier was detected. A paired-samples *t* test revealed a significant difference between the response initiation accuracy for self-associated match versus stranger-associated match trials, *t*(26) = 9.77, *p* < .001, *dz* = 1.88; self-associated match responses were initiated more quickly. Supporting the findings of the paired-samples *t* test analysis, a Wilcoxon signed-rank test determined that the median difference (.25) between response initiation accuracy (proportion correct) in the self-match (*Mdn* = .94) as compared with stranger-match condition (*Mdn* = .69) was statistically significant, *z* = 4.51, *p* < .001.

##### Sensitivity (signal detection) indices for response initiation

Next, *d*′ values for the self-related and stranger-related responses were compared. The difference sensitivity scores were normally distributed, but there was one outlier. A significant difference was found between the sensitivity index for self-related (*M* = 2.49, *SD* = 0.68) as compared with stranger-related response initiations (*M* = 1.50, *SD* = 0.60), *t*(26) = 7.62, *p* = .001. As expected, there was an advantage for self-related responses. A Wilcoxon signed-rank test supported these findings. The median difference (0.98) between *d*′ in the self-match (*Mdn* = 2.72) as compared with stranger-match condition (*Mdn* = 1.42) was statistically significant, *z* = 4.30, *p* < .001.

#### Movement execution

##### Movement time (MT)

Four nonextreme differences scores (Stranger – Self) outliers were detected. A paired-samples *t* test revealed a significant difference between the MTs on self-associated match versus stranger-associated match trials, *t*(26) = 9.53, *p* < .001, *dz* = 1.83; self-associated match responses were executed more quickly. Supporting the findings of the parametric test, a Wilcoxon signed-rank test determined that the median difference (93 ms) between MTs in the self-match (*Mdn* = 566 ms) as compared with stranger-match condition (*Mdn* = 657 ms) was statistically significant, *z* = 4.52, *p* < .001.

##### Movement response completion (proportion correct)

A paired-samples *t* test revealed a significant difference between the proportion of correctly completed movement responses in the self-associated as compared with the stranger-associated matched condition, *t*(26) = 10.62, *p* < .001, *dz* = 2.04.

#### Comparing the relative advantage for self in initiation and execution response stages

Next, we compared the self-advantage in RTs and MTs, and in proportion of correctly initiated and executed movement responses, to test whether the self-advantage was altered across the two-stage response. Normalized self-bias scores were calculated for RT, MT, and proportion of correctly initiated and completed movement responses (see Design section).

A paired-samples *t* test revealed a significant difference between the self-bias in RT (*M* = 0.11, *SD* = 0.06) and MT (*M* = 0.09, *SD* = 0.05), *t*(26) = 5.64, *p* < .001, *dz* = 1.09. The self-bias in RTs was greater than in MTs. A paired-samples *t* test also revealed that the self-advantage in the proportion of correctly completed movement responses (*M* = 0.26, *SD* = 0.14) was significantly greater than the self-bias in the proportion of correct response initiations (*M* = 0.15, *SD* = 0.09), *t*(26) = 5.58, *p* < .001, *dz* = 1.07. However, the distribution of the difference scores was only roughly approximately normally distributed and not symmetrical. Therefore, an exact sign test was additionally used in order to compare the self-bias in the proportion of correct response initiations with self-bias in the proportion of correctly executed movement responses. There was a statistically significant median difference (.08) between self-bias in the proportion of correctly completed movement responses (*Mdn* = .28) and self-bias in the proportion of correct response initiations (*Mdn* = .15), *z* = 4.62, *p* < .001. This result supported findings using the parametric test.

#### Relationship between the self-advantage in RT and MT

There were two nonextreme outliers in the MT self-bias index scores. With the outliers included, a strong positive correlation was found between the magnitude of the self-advantage in RT and MT, *r*(25) = .92, *p* < .001. With the outliers excluded, there was similarly a strong positive correlation between the magnitude of the self-advantage in RT and MT, *r*(23) = .87, *p* < .001. Small *n* correlations can, however, be unreliable (see Rousselet, 2021; Schönbrodt & Perugini, 2013). A Bayesian correlation analysis was also conducted, which further supported these findings. Bayes factors were calculated using the Bayesian-Correlation module of JASP (Version 0.12.2; JASP Team, 2020) and the JASP default prior. The Bayes factor in favour of the alternative model for the correlation between normalized RT and MT self-bias index scores across initiation and execution of responses was BF_10_ = 4.95e+8 indicating that there was ‘very strong’ or ‘decisive’ evidence for a correlation between RT and MT normalized self-bias index scores (Jeffreys, 1998; Raftery, 1995).

### Discussion

An advantage for responses to the self-associated stimuli in RT, MT, accuracy (proportion of accurately selected responses), and in the proportion of correctly completed movement responses was observed, consistent with the results of previous research (Desebrock et al., 2018; Sui et al., 2012). These findings reveal that the advantage for self in the execution of responses is not specific to the task response used by Desebrock et al. (2018) and is robust under conditions in which there is no visual feedback to guide participants’ responses. Importantly, our findings indicate that self-referential processing in the arm-movement responses does not reflect an explicit decisional response bias operating through visual-feedback-driven online control. The advantage for self in arm-movement responses, then, is not contingent on the modulation of visual-specific processing of hand and/or target position (Gallivan et al., 2018; Krüger & Hermsdörfer, 2019) and/or integration with proprioceptive information to estimate hand position (Scott, 2016). These findings also suggest that self-relevance can modulate movement responses (predominantly) driven by proprioceptive, kinaesthetic, and tactile information, consistent with Macrae et al. (2017), who found that self-relevance modulated either one or both of encoding and response execution in an identification task using button-press responses. The significant difference between normalized self-bias in the accurate initiation and execution stages of the response indicated that, once released, fewer stranger-associated as compared with self-associated movement responses successfully hit the target. Further implications of the differences and relationship between the relative advantage for self across the initiation and movement execution stages of the response are noted in the General Discussion.

A limitation of the present experiment was that movement responses were still effectively directed ‘toward’ the stimuli. Although responses did not visibly decrease the distance between the participant’s hand and the stimuli, simply ‘labelling’ a response as ‘toward’ has been argued to automatically assign it a positive valence, and through an affective S–R congruency between valence of the stimulus and motor response, movements can also be facilitated. According to the evaluative coding hypothesis (Eder & Hommel, 2013; Eder & Rothermund, 2008), the intentional affective evaluation of stimuli automatically activates a behavioural *goal* that, in turn, facilitates a correspondingly valenced motor response, irrespective of the distance from a self-representation (Phaf et al., 2014). Therefore, in Experiment 2, we further tested whether the approach–avoidance context impacted the movement response by directing task responses ‘away’ from both the stimuli and the participant’s body. As such, the task responses were labelled as ‘away’ (negative) as opposed to ‘toward’ (positive) in Desebrock et al. (2018). Therefore, if automatic affective evaluation or an intentional affective S–R mechanism do not *solely* account for the facilitation of self- relative to stranger-associated motor responses in Desebrock et al.’s study, again, an advantage for self-associated as compared with stranger-associated responses should be observed.

## Experiment 2

### Method

Effect sizes in the previous experiment and Desebrock et al.’s (2018) study were consistently large. However, if motivational orientation processes part-accounted for the advantage for self in response execution, then, once again, the effect size may be diminished. Therefore, in order to detect a medium-large-sized effect (*dz* = 0.70), with a probability of 1 − β = .80, and an alpha value of .05, a minimum sample size of 18 participants was required.

#### Participants

Twenty participants (five males, ages 18–40 years, mean age 22.20 ± 6.31 years) took part in Experiment 2. The data from five participants were excluded (two due to a technical issue, one for not following instructions, and two for scoring less than 55% accuracy in two or more conditions). The data from 15 participants (5 male, ages 18–35 years, mean age 21.73 ± 5.39 years) were included in the final analysis. The effect size detectable with 15 participants, an alpha value of .05, and a probability of 1 − β = .80 was *dz* = 0.78. (G*Power 3.1 program; Faul et al., 2009).

#### Apparatus and procedure

As in the procedure introduced by Desebrock et al. (2018), the participants held down two ‘home’ response-box buttons with their index fingers until a response was required. However, in contrast to Desebrock et al.’s study, the participants were instructed to execute a sideways motion task response, ‘away’ from themselves and the stimuli. The response box was positioned in between two PC QWERTY keyboards, and a response consisted of releasing the relevant RB button and moving the arm on the ipsilateral side out sideways along the horizontal, sagittal plane to press the relevant keyboard target key on the same side. The keyboard positioned to the right of the response box recorded MTs of ‘matching’ trial responses (executed using the right-hand to depress the key ‘z’), and the keyboard to the left of the response box recorded MTs of ‘mismatching’ trial responses (executed using the left hand to depress the ‘5’ key). The response box and keyboard target keys were aligned such that the right-hand RB key and target key and the left-hand RB key and target key were 13 cm apart, respectively. Following Desebrock et al., whose participants executed movement responses over the same travel distance, a movement response time limit of 1,250 ms was set (see Supplementary Materials for more information).

#### Data analysis

**Fig. 4:**
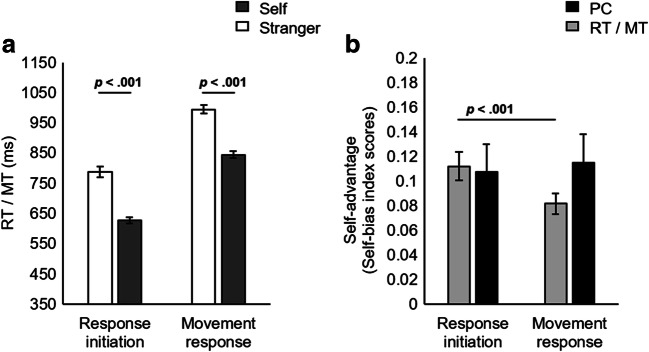
Experiment 2. **a** Mean RT (reaction time) and MT (movement time) as a function of association (self vs. stranger) in the shape-label matching trials. **b** Mean normalized self-bias index scores (magnitude of the self-advantage) in RT, MT, and proportion correct (PC) as a function of response stage (response initiation vs. movement response) in the shape-label matching trials. Error bars represent standard error

Only correct response initiations (RTs) were analyzed. RTs above or below 2.5 standard deviations from individual means were excluded, eliminating less than 2% (74) of the trials. Similarly, only correct movement responses (MTs) following a correct initiation response within 2.5 standard deviations of individual means were analyzed. MTs greater than 2.5 standard deviations above individual means were excluded, resulting in the elimination of less than 1% (48) of the trials. Overall, RT analyses were carried out using 87% of the trials, and MT analyses using 84%. Means and standard errors for self-associated and stranger-associated matching conditions are visualized in Fig. 4 (see Appendix, Table 2, for means and standard deviations of RT, MT, and proportion correct for response initiation and execution responses in match and mismatch trials).

### Results

Following Experiment 1, analysis of RT, MT, response initiation accuracy, and movement completion accuracy was made on the match-trial data. For tabulated match and mismatch trial data, see Appendix, Table 2.

#### Response initiation

##### Reaction-time (RT)

A paired-samples *t* test revealed a significant difference between the RTs for self-associated match versus stranger-associated match trials, *t*(14) = 8.98, *p* < .001, *dz* = 2.32; self-associated match responses were initiated more quickly.

##### Response initiation accuracy

One extreme outlier was detected. With the outlier included, a paired-samples *t* test revealed a significant difference between the response initiation accuracy for self-associated match versus stranger-associated match trials, *t*(14) = 5.48, *p* < .001, *dz* = 1.41; self-associated match responses were initiated more accurately. Supporting the findings of the *t* test, a sign test determined that the median difference (.16) between response initiation accuracy (proportion correct) in the self-match (*Mdn* = .96) as compared with stranger-match condition (*Mdn* = .76) was statistically significant, *z* = 3.62, *p* < .001. All 15 participants were more accurate in the self-associated as compared with stranger-associated match trials.

##### Sensitivity (signal detection) indices for response initiation

Next, indices of sensitivity (*d*′) were compared for the self-related and stranger-related responses. A significant difference was found between the sensitivity index for self-related (*M* = 2.86, *SD* = 0.49) as compared with stranger-related response initiations (*M* = 2.17, *SD* = 0.73), *t*(14) = 4.10, *p* = .001. In particular, there was an advantage for self-related responses.

#### Movement execution

##### Movement time (MT)

A paired-samples *t* test revealed a significant difference between the MTs on self-associated match versus stranger-associated match trials, *t*(14) = 9.31, *p* < .001, *dz* = 2.40; self-associated match responses were executed more quickly.

##### Movement response completion (proportion correct)

A paired samples *t* test revealed a significant difference between the proportion of correctly completed movement responses in the self-associated as compared with the stranger-associated matched condition, *t*(14) = 5.64, *p* < .001, *dz* = 1.46.

#### Comparing the relative advantage for self in initiation and execution response stages

As in Experiment 1, the self-advantage in RTs and MTs, and in the proportion of correctly initiated and executed movement responses, was compared to test whether the self-bias was altered across the two-stage response. A paired samples *t* test revealed a significant difference between the normalized self-bias in RT (*M* = 0.11, *SD* = 0.04) and MT (*M* = 0.08, *SD* = 0.03), *t*(14) = 7.85, *p* < .001, *dz* = 2.03. The self-bias in RTs was greater than in MTs.

The self-bias in the proportion of correct responses was then compared across the initiation (*M* = 0.11, *SD* = 0.09) and execution (*M* = 0.12, *SD* = 0.09) of the movement responses. The distribution of the difference scores was only roughly approximately normally distributed and not symmetrical. Therefore, an exact sign test was additionally used in order to compare the self-bias in the proportion of correct response initiations with self-bias in the proportion of correctly executed movement responses. There was no statistically significant median difference (.01) between self-bias in the proportion of correctly completed movement responses (*Mdn* = .09) and self-bias in the proportion of correct response initiations (*Mdn* = .09), *z* = 1.03, *p* = .30. A Bayesian Wilcoxon signed-rank test, however, indicated that evidence for the null model was inconclusive. Bayes factors were calculated using the Bayesian-T-tests/Wilcoxon signed-rank module of JASP (Version 0.12.2; JASP Team, 2020) and the JASP default prior. The Bayes factor in favour of the null model for normalized self-bias compared across the proportion of correct response initiations and correctly executed movement responses was BF_01_ = 0.94, indicating that there was ‘weak’ or ‘anecdotal’ evidence for the null model (Jeffreys, 1998; Raftery, 1995).

#### Relationship between the self-advantage in RT and MT

A strong positive correlation was found between the magnitude of the self-advantage in RT and MT, *r*(13) = .97, *p* < .001. A Bayesian correlation analysis was also conducted, which further supported these findings. Bayes factors were calculated using the Bayesian-Correlation module of JASP (Version 0.12.2; JASP Team, 2020) and the JASP default prior. The Bayes factor in favour of the alternative model for the correlation between normalized RT and MT self-bias index scores across initiation and execution of responses was BF_10_ = 3.111e+6, indicating that there was ‘very strong’ or ‘decisive’ evidence (Jeffreys, 1998; Raftery, 1995) for the correlation between RT and MT normalized self-bias index scores.

### Discussion

An advantage for responses to the self-associated stimuli in RT, MT, and accuracy (proportion of accurately-selected responses) was observed, along with more efficient movement responses (shorter MTs without compromising completion accuracy) in self-associated as compared with stranger-associated trials. Together with Experiment 1, these findings show that the advantage for self in arm movements is robust under multiple task conditions and is not specific to Desebrock et al.’s (2018) particular task response. Importantly, the results of Experiment 2 also revealed that the advantage for self in action is not wholly contingent on participants executing forward-motion arm-movement responses directed ‘toward’ the stimuli. In Experiment 2, self-associated arm-movement responses were executed ‘away’ from positively evaluated stimuli and the body, and, in stranger-associated responses, ‘away’ from more negatively evaluated stranger-associated stimuli, while visibly increasing the distance between the hand and the stimulus. Thus, according to both a distance-regulation and evaluative coding account of approach/avoidance action tendencies, self-associated responses should have been relatively disadvantaged, and stranger-associated responses potentially advantaged, as compared with the responses in Desebrock et al.’s task. If automatic or intentional affective S–R compatibility *solely* underpinned the advantage for self in movement responses in Desebrock et al.’s study, the advantage for self should have been extinguished, or the sign perhaps even reversed in Experiment 2, which was not the case. The possibility that the self-advantage may be reduced (i.e., that affective S–R compatibility may *part*-underpin the self-advantage) is explored further in a comparison of the self-advantage across experiments in the Supplementary Materials. Implications of the differences and relationship between the relative advantage for self across the initiation and movement execution stages of the response are also explored in the General Discussion.

## General discussion

Using an adaptation of Sui et al.’s (2012) matching task for investigating the Self-Prioritization Effect (SPE), the present study investigated whether self-relevance can modulate the initiation and execution of arm-movement responses, consistent with a multiple-stage influence (Humphreys & Sui, 2016; Sui & Humphreys, 2015). Specifically, we examined whether the self-advantage in the duration and accurate completion of arm-movement responses found in visual-feedback-driven arm-movements directed toward the stimuli (Desebrock et al., 2018) would be robust in ballistic movement without visual feedback (Experiment 1), and in avoidance movements, with responses directed away from the stimuli and participant’s body (Experiment 2). An advantage for self-associated movement responses (as well as their initiation) was observed in both Experiments 1 and 2. These findings suggest that the self-advantage in movement does not depend on affective S–R compatibility processes (Experiments 1 and 2), nor an explicit response bias operating through visual-feedback-driven execution processes (or an automatic modulation of these processes; Experiment 1). They further indicate that self-relevance can modulate movement responses (predominantly) driven by proprioceptive, kinaesthetic, and tactile information (Experiment 1). A preliminary analysis examining the self-advantage across experiments (provided in the Supplementary Materials) also suggested that the advantage for self in movement responses was not *part*-dependent on a modulation of visual-feedback driven and affective evaluation processes. The present study findings therefore support the contention that self-relevance in the matching task can modulate both the initiation and execution of movement responses, consistent with a multiple-stage influence (Humphreys & Sui, 2016; Sui & Humphreys, 2015).

### Accounting for the advantage for self in the motor stage

Only two other studies to date have considered the influence of self-relevance on the motor stage of responses in Sui et al.’s matching task (Desebrock et al., 2018; Janczyk et al., 2019). In a study aimed at pinpointing the locus of the SPE, Janczyk et al. (2019) found that the SPE reflects a modulation in central-stage processes, and, in a preliminary finding, that the motor stage (of the discrete key-press task responses) did not contribute to the SPE (Janczyk et al., 2019, Experiment 4). Other research has similarly found that effects of self-relevance may only be distinguished in later-stage, higher-order cognitive processes (Miyakoshi et al., 2007; Siebold et al., 2015; Stein et al., 2016). In contrast, the present study and Desebrock et al. (2018) found a self-advantage in both the initiation and execution of arm-movement responses, consistent with a multiple-stage influence (Humphreys & Sui, 2016; Liu et al., 2016; Macrae et al., 2017; Sui & Humphreys, 2015).

**Fig. 5:**
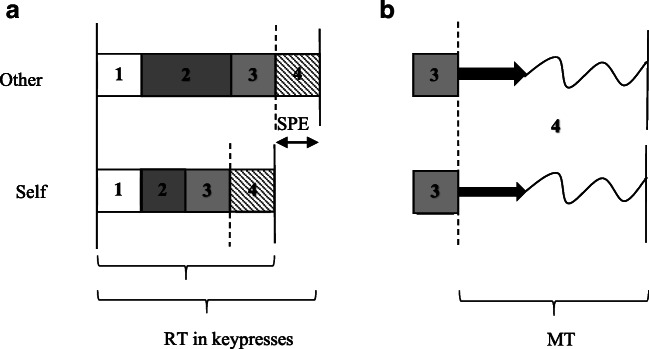
**a** Representation of Janczyk et al.’s (2019) conception of where the SPE arises in key-press responses in Sui et al.’s (2012) matching task. Other = other-associated matched trial responses. Self = self-associated matched trial responses. RT denotes the interval between stimulus onset to response completion in key-press responses and includes (1) *perceptual,* (2) *central,* (3) *motor-specific preparatory,* and (4) *overt movement execution* stage processes. NB: In the present study, RT denotes the interval between stimulus onset and button release (i.e., 1–3). Response execution processes include both (Box 3) preparatory motor activity and (Box 4) online correction control (Allsop et al., 2017; Khan et al., 2006). Dotted lines represent the moment at which the overt movement begins. Solid lines represent stimulus onset or response completion. **b** Representation of the motor stage (response execution processes). MT denotes the interval from movement onset (button release) to response completion in arm-movement responses (in the present study). (NB: MT in key presses is subsumed within RT.) The one-way arrows represent the initial impulse of the overt movement reflecting motor planning processes that occur prior to movement onset. The wavy lines represent online correction processes that occur post-movement-onset during movement execution (Allsop et al., 2017; Khan et al., 2006). (NB: In speeded tasks, online correction of discrete key-press responses does not typically occur; Oulasvirta et al., 2018). Shown here is the hypothetical case in which there is no modulation of response execution processes by self-relevance, with movement preparation yoked to the initiation of the overt movement. (NB: That movement preparation and initiation are yoked is an implicit assumption of Janczyk et al.’s study and traditional stage-model theory; cf. Haith et al., 2016.)

Janczyk et al.’s (2019) findings are in line with earlier research (e.g., see Donders, 1969; Fitts, 1954; Fitts & Radford, 1966; Frowein & Sanders, 1978; Glencross, 1976; Posner, 2005; Sternberg, 1969) which found that the motor-stage of a *short rapid movement to a target* is influenced by target, but not stimulus-related features. Similarly, more recent optimal feedback control (OFC) models hold that a control policy that minimizes the cost of the movement in terms of effort, inaccuracy, and regularization determines movement planning and execution (cf. Gallivan et al., 2018; Yeo et al., 2016). Both lines of research predict that the speeded overt movement responses in the matching trials (that have the same movement goal and use the same effector) should be equivalent across self and stranger conditions, as Janczyk et al. found using key-press task responses. A schematic illustration of what Janczyk et al.’s proposition entails is shown in Fig. 5. Note how self-associated responses have an advantage through Stage 2 (central processing), while the perceptual, motor-specific preparatory, and overt movement execution stages are equivalent across self-associated and stranger-associated responses.

In contrast to the scenario depicted in Fig. 5, the present study found that the overt movement (Phase 4; Fig. 5b) was modulated in Sui et al.’s (2012) task. Furthermore, the advantage for self-associated arm-movement responses did not depend on a modulation of online visual feedback-driven processes (either reflecting an explicit decisional response bias, or an automatic modulation of these processes), nor was (solely) driven by automatic affective evaluation and S–R compatibility processes. What mechanism(s) can therefore account for the modulation of execution processes by self-relevance?

#### Distinguishing between potential accounts of the movement self-advantage

Three potential accounts of the present findings are: (1) the advantage for self in central processes directly drives the advantage for self in the motor stage; (2) the advantage for self in central processes and the motor stage are independently driven by a third factor; (3) the advantage for self in central processes and the advantage for self in the motor stage are independent, influenced by distinct factors.^3^

It has been suggested that enhanced attention to self-associated stimuli increases certainty in decision-making processes pertaining to those stimuli (Liu et al., 2016; Sui & Humphreys, 2015), coupling the two processes (see also Macrae et al., 2018). In an ERP study using a face cueing and discrimination task, Liu et al. (2016) found a correlation between the self-bias in attention (indexed by the N1 component) and decision-making processes (indexed by P3). A correlation was also found between the self-advantage in RT and MT in the present study. Could the advantage for self in central processes be similarly coupled to the motor stage of responses in the matching task? Movement dynamics (i.e., speed, amplitude, duration; Berret et al., 2018), which influence MT, are driven by an ‘urgency’ or ‘movement vigor/vigour’^4^ signal (Dudman & Krakauer, 2016; Panigrahi et al., 2015; Reppert et al., 2018; Turner & Desmurget, 2010), which is thought to arise in the basal ganglia (Thura et al., 2014; Thura & Cisek, 2017). Similarly, in decisional processes, an urgency signal speeds up RTs (Thura & Cisek, 2017). Speiser et al. (2017) found that, under certain conditions, the SAT mechanism in decisional processes can speed up motor-specific preparation (reflected in a faster build-up of EMG-activity), and produce faster movements (but increased overt errors). Indeed, until recently, it was thought that decision urgency and movement vigour may constitute a unique ‘invigoration’ signal, influencing latencies in both decisional and motor processes (see Reynaud et al., 2020). In this account, the self-advantage in RT could drive the self-advantage in MT through a shared invigoration signal. However, recent work has shown that decisional urgency and movement vigour are actually independent (albeit interacting) signals (Reynaud et al., 2020). In line with this, a recent study by Barton et al. (2020) found that self-mug ownership shortened both RT and MT in approach movements and only RT in (participant-body-directed) avoidance movements. Therefore, the finding of the present study that participants with a larger magnitude self-advantage in decisional processes also had a larger magnitude self-advantage in execution processes would necessarily reflect the operation of two independent signals. Their interaction, however, must be coordinated by a third factor (Reynaud et al., 2020; a potential candidate in relation to self-referential and stranger-referential processing is discussed below). This would also counter the possibility that the differences in the magnitude of normalized self-bias in RT and MT found across initiation and execution were an artefact of a single decisional urgency signal—namely, that the decisional-urgency-driven movement simply interacts with the mechanics of the movement in such a way that reduces the self-advantage seen in RT. Furthermore, if this were the case, one would also expect the decisional urgency signal to interact with the differential mechanics of the task responses across experiments, but the magnitude of normalized self-bias in MT did not change across experiments.

Following Reynaud et al. (2020), and as noted above, the self-advantage in RT and MT must be coordinated by a third factor. One potential candidate is the vmPFC (necessarily through connections with the basal ganglia; Reynaud et al., 2020). It has been proposed that activity in the vmPFC (thought to house a self-representation; Humphreys & Sui, 2016) is rapidly activated by self-relevance and modulates responses by functionally coupling with distinct domain-specific regions associated with different components of the self (Sui & Humphreys, 2015). For example, functional connectivity between the vmPFC and an area linked to social attention (pSTS) and between the vmPFC and a classic WM region (frontoparietal cortex) have been demonstrated to underpin self-prioritization in the matching task (Sui et al., 2013; Yankouskaya et al., 2017) and a spatial WM task (Yin et al., 2021), respectively. Such a coupling may also extend to motor-linked regions, perhaps influencing both latencies and movement completion accuracy across self-associated and stranger-associated movement responses.

Thus far, the focus of the discussion has been on the self-advantage in RT and MT. The present study also found that a higher proportion of movement responses in self-related as compared with stranger-related matching trials were correctly completed (Experiment 1 and Desebrock et al.’s, 2018, study), or that MTs were shorter without sacrificing movement completion accuracy (Experiment 2). In this sense, movement responses in self-associated matching trials were more efficient. As noted, speeding up EMG-activity results in faster movements, but also increases overt errors (Speiser et al., 2017). Other work has also shown that the urgency signal does not influence endpoint accuracy (Reppert et al., 2018). Modulations of urgency/movement vigour could therefore not solely account for the self-advantage in the movement responses. Could self-prioritization in central processes account for the self-advantage in more efficiently completed movement responses?

Central processes include selection into and switching between items in working memory (WM; Janczyk, 2017), and response selection (Janczyk & Kunde, 2020; Welford, 1952). As noted in the Introduction, Frings and Wentura’s (2014) study suggests that a representation of the movement response (e.g., internal verbal description/motor imagery) may be accessed more efficiently in self-associated trials. Thus, faster and more accurate *selection* of action representations in line with the movement goal may contribute to the advantage for self in RT and decisional accuracy. However, such a mechanism does not account for the modulation of movement execution across self-associated and stranger-associated trials (which used the same effector and target).

Self-associations established in the matching task can also modulate working memory (WM). Using a delayed match-to-sample spatial task, Yin et al. (2019) found self-representations were afforded superior maintenance in WM, through internal attentional processes. As they state, “Prioritization of information in [WM] . . . allows us to temporarily keep information in mind for additional cognitive processing and the guidance of actions” (p. 3). Indeed, visual imagery maintained by working memory can guide action (Ede et al., 2019). The visual image of the target and its location may be formed more clearly in self-associated match trials, or just held in mind throughout the task (despite attention being diverted to onscreen stimuli) and more efficiently accessed in self-associated match trials. This putative mechanism could be involved in the self-advantage in movement responses in Experiment 2 and Desebrock et al.’s (2018) study. However, it would not explain the self-advantage in movement in Experiment 1, where no visual feedback was available pertaining to the target or participant’s hands. Alternatively, proprioceptive/kinaesthetic/tactile imagery may be involved in the self-advantage in movement responses. Indeed, in Experiment 1, movement planning relied on proprioceptive, kinaesthetic, and tactile feedback. How such imagery might differentially influence the execution of self-associated and stranger-associated match-trial movement responses, however, falls outside the scope of the present discussion.

In the Supplementary Materials, we suggest that differential movement planning may underpin differences in the execution of self-associated as compared with stranger-associated movement responses based on the current findings and a preliminary analysis comparing the self-advantage across Experiments 1, 2, and Desebrock et al.’s (2018) study. Across experiments, we found that the magnitude of the self-advantage in correctly completed movement responses was significantly greater in Experiment 1. Where self-associated movements remained consistent across all response types, stranger-associated movements appeared to be further disadvantaged by the response type of Experiment 1 (i.e., a ballistic movement response with no visual feedback available). In other words, the self-advantage interacted with movement response type (although, see Supplementary Materials for a cautionary note regarding this finding). In summary, therefore, we can conclude the following: the self-advantage in the proportion of correctly completed movement responses also emerges in ballistic movement (reflecting movement planning; Glover, 2004; Khan et al., 2006) and does not depend on visual information about the target or participant’s hands in the planning or execution of ballistic movement responses; stranger-associated, as compared with self-associated, movement responses were less efficiently completed across all experiments (movement response types), and were apparently further disadvantaged when generating ballistic movements without visual information as compared with nonballistic movements using visual feedback. If differences in the movement vigour signal cannot (solely) account for the self-advantage in movement responses, we therefore speculate that the self-advantage may (also) interact with movement planning.

It is possible that despite planning the same goal-directed movement in the self-associated and stranger-associated conditions (i.e., the goal to hit the same target key in the matching condition), a distinct sensorimotor control policy may be engaged across conditions. The stranger-related control policy may be more or less compatible with the type of movement required. Compatibility may be particularly relevant in a fast ballistic movement to a target that is executed without visual feedback before during or after execution—in other words, one that cannot be initially planned, guided, recalibrated using exogenous visual information. (As suggested above, it may be that distinct neural circuitry underpins self as compared with stranger-associated movements, as has been found to underpin self and stranger responses in the standard matching task; Sui et al., 2013). In a feedforward model of sensorimotor control (Yeo et al., 2016), it has been proposed that in addition to planning based on sensory information, movements are also planned in terms of the *consequences* of the movement on sensory feedback. This necessarily implicates a feedforward component (Yeo et al., 2016)^5^ which may draw on internal representations. Such a notion is in line with a growing recognition in the sensorimotor control literature that cognitive factors can influence movement selection, planning, and control at multiple levels of human information processing (Gallivan et al., 2018). As such, this raises the interesting question of whether such internal representations and feedforward components may be susceptible to influence by self-relevance. In the context of the matching task (Desebrock et al., 2018; Sui et al., 2012), self-prioritization may interact with feedforward components in terms of the anticipation of associated real-world consequences for self-associated versus other-associated movements (which may, for example, differentially use visual feedback in a manner that may be more or less compatible with the requisite task response; cf. Janczyk & Kunde, 2020, for effect anticipation processes in response selection).

### Limitations of the present study

The present study cannot systematically determine whether the self-advantage interacts with visual feedback and whether self-relevance could also modulate online processes in nonballistic movements without visual feedback. However, we can conclude that the self-advantage does not depend on visual information in either the planning or execution of movement responses, nor on a modulation of non-ballistic movement, and that self-relevance can modulate ballistic movement (reflecting movement planning; Glover, 2004; Khan et al., 2006) in the absence of visual information, as well as influencing movement generally, in multiple directions. These findings also speak to the versatility of self-associated as compared with stranger-associated movement responses.

A limitation as well as strength of the present study, however, is the use of the matching task. It provides the opportunity to examine self-related and stranger-related processing in responses using the same effector and movement goal. On the other hand, Golubickis et al. (2017) found a response bias was operating in matching trials. Binding two pieces of information (i.e., the shape and label) in the present study may therefore have increased effect sizes. Notably, however, the SPE also arises in *identification* tasks (e.g., Janczyk et al., 2019, Experiment 3). When and how self-prioritization arises can be task-dependent (Caughey et al., 2020; Falbén et al., 2019; Golubickis et al., 2017), so future studies are needed to test whether the self-advantage in movement responses arises in other decisional environments (cf. categorization tasks; Caughey et al., 2020; Falbén et al., 2019). More explicit self-related and other-related judgments may be impacted by the approach-avoidance context, for example.

The present study provides further support that the execution of arm-movement responses in Sui et al.’s (2012) task can be modulated by self-relevance. Richer insights could be gained in future studies by systematically investigating *how* self-relevance modulates the motor stage, which could not be determined by the present study. For example, using kinematic analysis to measure directional error in the initial impulse (Khan et al., 2006) could provide insight into differences in the quality of movement planning processes in self- as compared with stranger-associated responses.

One further note is that the interpretative framework of the present study assumes that perceptual, central, and motor stages of cognitive processing can be distinguished (in line with classical cognitive psychology and much research in the self-prioritization literature, e.g., Janczyk et al., 2019). However, neuroscience research has shown that decisional and motor processes do not necessarily unfold in a serial manner (Kaufman et al., 2015). For example, where activity in the motor cortex once served as an index for purely motor processes (for example, in ERP studies), this preparatory ‘motor’ activity can reflect, for example, vacillation or hesitation during the decisional process (Kaufman et al., 2015). Responses can be selected, unselected, and reselected before a commitment to a response is made. Although vacillation, indecision, and hesitation can all influence RT duration, interpretation of the present study findings can accommodate this possibility. Execution of the movement once a commitment to a response has been made was measured separately from response selection processes. The button being released in the present study’s bimanual task responses indicated the motor decision (which target), and so response selection processes ended prior to movement onset (Rubichi & Pellicano, 2004; Scorolli et al., 2015). We also ruled out that explicit decisional response biases operating through visual-feedback-driven processes underpin the self-advantage. Furthermore, although ‘changes of mind’ can arise in movement responses, Kaufman et al. (2015) observed in their study that these internal processes were specific to free choice trials, and never observed in forced-choice trials. There are other developments in neuroscience, however, such as the more radical approaches of 4E (embodied, embedded, enactive, and extended) cognition and predictive coding theories based on the free energy principle, which do not share the present study assumptions about the architecture of cognition (see Newen et al., 2018). Future studies could examine self-referential versus other-referential processing in the context of these alternative frameworks to provide new insights into self-referential processing in action.

## Conclusion

The present study examined whether self-relevance can modulate the motor stage of responses in Desebrock et al.’s (2018) adaptation of Sui et al.’s (2012) matching task. In Experiment 1, visual feedback was occluded and a ballistic task response used, while in Experiment 2, responses were directed away from the stimuli and the participant’s body. The advantage for self in the initiation and execution of arm-movement responses emerged in both experiments. These findings indicated that the self-advantage in arm-movement responses does not depend on affective S–R compatibility processes, nor on an explicit response bias operating through visual-feedback-driven execution processes. They support the view that self-relevance in Sui et al.’s matching task has a multiple-stage influence (Humphreys & Sui, 2016; Sui & Humphreys, 2015), countering the suggestion that effects of self-relevance arise only in higher-order cognitive or central-stage processes (e.g., Janczyk et al., 2019).

### Supplementary Information

ESM 1 (DOCX 31.9 kb)

## Appendix

**Table 1:** Mean reaction times (RT) and movement times (MT) in ms and proportion of correctly initiated (response initiation PC) and correctly completed (movement completion PC) movement responses, with standard deviations, as a function of association (self vs. stranger) and match condition (matched vs. mismatched) in Experiment 1

Performance indices		Match condition	
		Matched	Mismatched
RT	Self	554 (59)	673 (55)
	Stranger	694 (69)	681 (50)
MT	Self	542 (53)	647 (32)
	Stranger	646 (43)	651 (34)
Response initiation PC	Self	0.93 (0.04)	0.80 (0.14)
	Stranger	0.70 (0.11)	0.82 (0.09)
Movement completion PC	Self	0.91 (0.07)	0.70 (0.16)
	Stranger	0.55 (0.15)	0.69 (0.13)

*Note.* Standard deviations appear within parentheses. PC = proportion correct.

**Table 2:** Reaction times (RT) and movement times (MT) in ms and proportion of correctly initiated (response initiation PC) and correctly completed (movement completion PC) movement responses, with standard deviations, as a function of association (self vs. stranger) and match condition (matched vs. mismatched) in Experiment 2

Performance indices		Match condition	
		Matched	Mismatched
RT	Self	627 (40)	754 (46)
	Stranger	787 (69)	744 (44)
MT	Self	845 (47)	958 (52)
	Stranger	995 (55)	953 (56)
Response initiation PC	Self	0.95 (0.03)	0.87 (0.07)
	Stranger	0.77 (0.12)	0.90 (0.05)
Movement completion PC	Self	0.93 (0.03)	0.82 (0.08)
	Stranger	0.75 (0.13)	0.87 (0.06)

*Note*. Standard deviations appear within parentheses. PC = proportion correct.

## Footnotes

1 Movement *initiation* refers to the information processing that occurs between stimulus onset and onset of the overt movement response, and movement *execution* refers to the overt movement response.

2 Central-stage processes include encoding into short-term memory (Jolicoeur & Dell’Acqua, 1998), selection into and switching between items in working memory (WM; Janczyk, 2017), and response selection (Janczyk & Kunde, 2020; Welford, 1952).

3 We note that much previous work has found that a modulation in perceptual processes (also) underpins self-prioritization (Humphreys & Sui, 2016; Sui et al., 2012). The potential mechanisms by which a perceptual modulation in Sui et al.’s (2012) task could influence the movement response are discussed in Desebrock et al. (2018).

4 Movement vigour (or vigor; Dudman & Krakauer, 2016; Panigrahi et al., 2015; Reppert et al., 2018; Turner & Desmurget, 2010) refers to the dynamics of motor performance, namely to “the interplay between amplitude, speed, duration or frequency of movements” (Berret et al., 2018, p. 1) and is modulated by an urgency signal that is thought to arise in the basal ganglia (Reynaud et al., 2020; Thura et al., 2014).

5 Yeo et al. (2016) argue that sensorimotor control involves a feedforward component. Visual and proprioceptive noise on the sensory input associated with limb position varies with the state of the body. Therefore, costs not only include accuracy and energy expenditure, but also the sensory consequences of the movement. “[Planning] has to take into account the consequences of the movement in shaping the upcoming sensory afferents, which will be used to guide the movement” (p. 2). This necessarily implicates a feedforward component.
