# Supplementary material for: The Self-Prioritization Effect: Self-referential processing in movement highlights modulation at multiple stages
Source: Atten Percept Psychophys. 2021 Apr 16;83(6):2656–74. doi: 10.3758/s13414-021-02295-0 (PMC8302500; doi:10.3758/s13414-021-02295-0)
Supplement: Supplementary file 1 — (DOCX 31.9 kb) [file 13414_2021_2295_MOESM1_ESM.docx]

***Supplementary materials***

*Comparing the advantage for self in arm-movement responses across experiments*

**Table 3**

Mean Reaction Times (RTs) and Movement Times (MTs) in ms and proportion of correctly- initiated (Response initiation PC) and correctly-completed (Movement completion PC) movement responses, with standard deviations, as a Function of Association (self vs. stranger) in the Match condition for *Experiments A1*, *1*, and *2*.

|  | Experiment A1*  (Visual feedback, responses directed toward the stimuli) | | Experiment 1  (Visual feedback occluded, responses directed toward the stimuli) | | Experiment 2  (Visual feedback, responses directed away from stimuli and participants’ body) | |
| --- | --- | --- | --- | --- | --- | --- |
| Association | Self | Stranger | Self | Stranger | Self | Stranger |
| RT | 635 (62) | 782 (66) | 554 (59) | 694 (69) | 627 (40) | 787 (69) |
| MT | 797 (60) | 931 (70) | 542 (53) | 646 (43) | 845 (47) | 996 (55) |
| R. PC | 0.96 (0.04) | 0.89 (0.09) | 0.93 (0.04) | 0.70 (0.11) | 0.95 (0.03) | 0.77 (0.12) |
| M. PC | 0.96 (0.03) | 0.79 (0.11) | 0.91 (0.07) | 0.55 (0.15) | 0.93 (0.03) | 0.75 (0.13) |

Note. Standard deviations appear within parentheses. R. = Response initiation; M. = Movement-completion; PC = proportion correct. *A new sub-set of data taken from a larger data set on which separate analyses were carried out and published in Desebrock et al. (2018). See main text.

The findings of Experiments 1 and 2 indicated that the advantage for self in movement responses is not solely contingent on a modulation of visual-feedback driven and affective evaluation processes. However the two experiments did not rule out that the advantage for self may be part-dependent on a modulation of visual-feedback driven and affective evaluation processes. Therefore, a preliminary analysis comparing the magnitude of the self-advantage in RT, MT, and the proportion of correctly-completed movement responses was made across Experiments 1, 2 and Desebrock et al.’s (2018) study. The experimental set-up in Desebrock et al.’s study was identical to that of Experiment 2 in the present study, except that movement responses were made toward the onscreen stimuli. In order to compare the findings of the present study with those of Desebrock et al.’s study, a sub-sample of participants from Desebrock et al.’s (2018) data set was used. These were the 17 participants that matched the task conditions of the current study experiments i.e. all the participants in Desebrock et al.’s study who executed match responses with their right-hand in the first session. (NB Desebrock et al., 2018, used a two-session experimental design across which dominant/non-dominant-hand match-trial responses were counterbalanced). This sub-sample and subsequent analyses of this data will hereafter be referred to as Experiment A1. The RT response time-limit was the same across all experiments. However, one caveat regarding the comparison is that a shorter MT response time-limit was used in Experiment 1. The shorter time limit was necessary to accommodate the much reduced travel distance in Experiment 1, to ‘push the system’ equivalently across tasks, and prevent participants using a non-ballistic or non-aiming response (e.g. where participants could feel around for the target button) and performance would have been at ceiling. Hence the following analysis should be considered preliminary in nature. We hypothesized that if the advantage for self was reduced in Experiments 1 and 2 as compared with Experiment A1*,* this would suggest a part-dependence on one or more of the above processes specific to Desebrock et al.’s task response.

A one-way ANOVA on normalised self-bias index scores (see Design) revealed no significant difference in the normalized RT self-bias scores across Experiment A1 (*N* = 17; *M* = 0.10, *SD* = 0.04), Experiment 1 (*N* = 27; *M* = 0.11, *SD* = 0.05), and Experiment 2 (*N* = 15; *M* = 0.11, *SD* = 0.04), *p* = .86. Similarly, ANOVA on the normalized MT self-bias scores across Experiment A1 (*N* = 17; *M* = 0.08, *SD* = 0.03), Experiment 1 (*N* = 27; *M* = 0.09, *SD* = 0.05), and Experiment 2 (*N* = 15; *M* = 0.08, *SD* = 0.03), *p* = .70, revealed no significant difference. In contrast, the self-bias in the proportion of correctly-completed movement responses was significantly different across Experiment A1 (*N* = 17; *M* = 0.10, *SD* = 0.07), Experiment 1 (*N* = 27; *M* = 0.26, *SD* = 0.14), and Experiment 2 (*N* = 15; *M* = 0.12, *SD* = 0.09), Welch’s *F*(2, 34.95) = 13.08, *p* < .001. Games-Howell post hoc analysis revealed a statistically-significant difference between Experiments A1 and 1, *p* < .001, and Experiments 1 and 2, *p* = .001, and no significant difference between Experiments A1 and 2, *p* = .92 (see Figure 3). Self-bias in the proportion of correctly-completed movement responses was significantly greater in Experiment 1.

**Figure 6.** Comparison of the normalised self-bias (magnitude of the self-advantage) in the proportion of correctly-completed movement responses across Experiments A1 and Experiments 1 and 2 of the present study [Experiment A1 was a sub-sample from Desebrock et al.'s (2018) data, see main text for details]. Error bars represent standard error. **p* =.001, ***p* <.001.

Bayesian analyses indicated ‘positive’ or ‘substantial’ evidence for the null effects (Jeffreys, 1998; Raftery, 1995). Specifically, Bayes factors were calculated using the Bayesian-ANOVA module of JASP version 0.12.2 (JASP Team, 2020) and the JASP default prior. The Bayes factor in favour of the null model for the normalised RT self-bias across experiments was BF_01_ = 6.34, and for the normalised MT self-bias across experiments was BF_01_ = 5.42.

These preliminary findings suggested that the advantage for self in the movement responses was not part-contingent on visual-feedback-driven processes or motivational processes in affective S-R compatibility. Furthermore, the magnitude of the self-advantage in the proportion of correctly-completed arm-responses was significantly greater in Experiment 1, than in either Experiment 2 or Experiment A1: There was a substantial drop in the proportion of correctly-completed movement responses in the stranger-associated condition. This suggested that the advantage for self in movement may also interact with the type of task-response.

**Interpreting the increased self-advantage in ballistic movement without visual feedback**

The present study could not determine whether the preliminary finding of a significantly greater self-advantage in Experiment 1, driven by a disadvantage for stranger-associated movements, was due to the removal of visual feedback or the ballistic response (as removal of visual feedback and the ballistic response constituted a two-factor difference between Experiment 1 and Desebrock et al.’s task response). However, making a short ballistic arm-movement response to a target over a 6cm travel distance should be an easy task (with which participants indeed did not have difficulty when making movement responses in self-associated trials). Imagining everyday examples, it is easy to intuit that performance should be near ceiling in such a task (although, in everyday examples, visual information is almost always involved). We may then claim that whether or not visual feedback was available should make no difference to responses (in contrast, removing visual feedback from Experiment 2 and Desebrock et al.’s tasks produced a floor effect). The easier task response of Experiment 1 did not require visual feedback for successful completion. However, if the removal of visual feedback made no difference, then then one would expect the task to be ‘easy’ for stranger- as well as self-associated responses. Stranger-associated responses in Experiment 1 should have somewhat closed the gap and reduced the self-advantage in movement-completion accuracy, or perhaps even extinguished it due to a ceiling effect. In ballistic movement responses where visual feedback *is* available (albeit seemingly not required for response completion), movements can be planned and re-calibrated throughout the task using visual information: Visual information about the participant’s hand and target can be gained through peripheral vision, or through direct-line vision at intervals during and between trials/blocks. In Experiment 1, this was not available. It may be that the finding that self-bias in movement-completion accuracy significantly increased in Experiment 1 reflects an interaction of the self-advantage with the availability of visual information, specifically in movement planning (based on the assumption that ballistic movements reflect movement planning not online correction processes). The present study cannot systematically determine whether the self-advantage interacts with visual feedback and whether self-relevance could also modulate online processes in non-ballistic movements without visual feedback. However, we can conclude that the self-advantage does not depend on visual information in either the planning or execution of movement responses, nor on a modulation of non-ballistic movement, and it can modulate ballistic movement (reflecting movement planning; Glover, 2004; Khan et al., 2006) in the absence of visual information. These findings also speak to the versatility of self- as compared with stranger-associated movement responses.
